# Notch-1 Signaling Regulates Microglia Activation via NF-κB Pathway after Hypoxic Exposure *In Vivo* and *In Vitro*

**DOI:** 10.1371/journal.pone.0078439

**Published:** 2013-11-06

**Authors:** Linli Yao, Enci Mary Kan, Charanjit Kaur, S. Thameem Dheen, Aijun Hao, Jia Lu, Eng-Ang Ling

**Affiliations:** 1 Key Laboratory of the Ministry of Education for Experimental Teratology, Shandong Provincial Key Laboratory of Mental Disorders, Department of Histology and Embryology, Shandong University School of Medicine, Jinan, Shandong, China; 2 Combat Casualty Care, Defence Medical and Environmental Research Institute, DSO National Laboratories, Singapore, Singapore; 3 Department of Anatomy, Yong Loo Lin School of Medicine, National University of Singapore, Singapore, Singapore; Temple University School of Medicine, United States of America

## Abstract

Neuroinflammation mediated by the activated microglia is suggested to play a pivotal role in the pathogenesis of hypoxic brain injury; however, the underlying mechanism of microglia activation remains unclear. Here, we show that the canonical Notch signaling orchestrates microglia activation after hypoxic exposure which is closely associated with multiple pathological situations of the brain. Notch-1 and Delta-1 expression in primary microglia and BV-2 microglial cells was significantly elevated after hypoxia. Hypoxia-induced activation of Notch signaling was further confirmed by the concomitant increase in the expression and translocation of intracellular Notch receptor domain (NICD), together with RBP-Jκ and target gene Hes-1 expression. Chemical inhibition of Notch signaling with N-[N-(3,5-difluorophenacetyl)-1-alany1- S-phenyglycine t-butyl ester (DAPT), a γ-secretase inhibitor, effectively reduced hypoxia-induced upregulated expression of most inflammatory mediators. Notch inhibition also reduced NF-κB/p65 expression and translocation. Remarkably, Notch inhibition suppressed expression of TLR4/MyD88/TRAF6 pathways. *In vivo*, Notch signaling expression and activation in microglia were observed in the cerebrum of postnatal rats after hypoxic injury. Most interestingly, hypoxia-induced upregulation of NF-κB immunoexpression in microglia was prevented when the rats were given DAPT pretreatment underscoring the interrelationship between Notch signaling and NF-κB pathways. Taken together, we conclude that Notch signaling is involved in regulating microglia activation after hypoxia partly through the cross talk between TLR4/MyD88/TRAF6/NF-κB pathways. Therefore, Notch signaling may serve as a prospective target for inhibition of microglia activation known to be implicated in brain damage in the developing brain.

## Introduction

Notch signaling pathway is one of the most conserved pathways with versatility in function [1]–[3]. Mammalian canonical Notch signaling is activated when either the Delta or the Jagged ligands bind to one of four Notch receptors, thus resulting in a two-step proteolytic cleavage by the A Disintegrin And Metalloproteinase and γ-secretase, which releases the intracellular Notch receptor domain (NICD) from the membrane [4]–[7]. NICD then translocates to the nucleus, where it associates with the transcription factor Recombining binding protein suppressor of hairless (RBP-Jκ) and other modulators to regulate a variety of cellular events, such as cell proliferation and differentiation [8], [9].

In the central nervous system (CNS), the Notch signaling pathway is prominent among processes known to regulate normal development mainly on the neural progenitor cells, neurons, oligodendrocytes and astrocytes [10]–[14]. Recent evidence suggests that Notch may also play an important role in regulating the responsiveness of immune cells to stimulation and infection [15]–[19]. Microglia is one of the most important immune cells in the CNS and partakes in diverse roles within the CNS, namely normal brain development and inflammatory diseases. The involvement of the Notch signaling pathway in microglia has only recently been reported, suggesting a putative role in mediating microglial maturation and activation [15], [20]–[22]. We have reported in previous studies that Notch signaling modulates the expression of proinflammatory cytokines and nitric oxide (NO) in activated microglia after lipopolysaccharide (LPS) stimulation [15], [20]. These observations suggest that Notch activation in microglia is induced during CNS inflammation and may serve to regulate microglia function and pathophysiology of neuroinflammatory diseases.

It is well documented that hypoxia is linked to different neurodevelopmental diseases [23]–[28]. In view of this, we have used in this study *in vitro* hypoxia models of both primary cultures of microglia and BV-2 cell line and an *in vivo* experimental rodent model with postnatal hypoxic exposure to investigate the role and mechanism of Notch signaling in neuroinflammation in the hypoxic developing brain. Although there are many Notch receptors and ligands that are all worthy of investigation, we have concentrated on the Notch-1 receptor and Notch ligand Delta-1 as we have found that Notch-1 was most significantly changed in our previous study investigating Notch signaling expression in microglia after LPS stimulation [20]. Hence, we felt it appropriate to concentrate on investigating Notch-1 expression in hypoxic microglia in this study. In addition, N-[N-(3,5-difluorophenacetyl)-1-alany1]-S-phenyglycine t-butyl ester (DAPT), a γ-secretase inhibitor, which can efficiently block the γ-secretase complex, was applied to investigate the response of the Notch inhibition.

## Materials and Methods

### Hypoxia treatment of postnatal rats

One-day-old postnatal rats (n = 10) were exposed to hypoxia by placing them in a chamber (Model MCO 18 M; SanyoBiomedical Electrical Co, Tokyo, Japan) filled with a gas mixture of 5% O_2_ and 95% N_2_ for 2 h. The rats were then allowed to recover under normoxic conditions for 3 and 7 d before sacrifice (n = 3 per time point); another group of 6 rats were kept outside the chamber and used as age-matched controls.

There was no differentiation between sexes and animals were randomized into control, and hypoxia groups. All hypoxic rats survived hypoxia treatment. The hypoxic rats were observed to suffer from severe cyanosis immediately after hypoxia and observed to recover after a few hours. Immediately after hypoxia, the rats were returned to their mother. Neonatal rats were accepted back by their mothers. No observable difference in size, body weight and general behaviour could be seen 3 days after hypoxia.

Postnatal rats (n = 3) were given a single intraperitoneal injection of DAPT (10 mg/kg Sigma-Aldrich, St. Louis, MO; Cat. No. D5942), a γ-secretase inhibitor, 1 h before hypoxia to investigate the effect of Notch blockade *in vivo* [29], [30]. Control rats were subjected to hypoxia without DAPT pretreatment (n = 3).

The study was approved by the Institutional Animal Care and Use Committee, National University of Singapore (IACUC no: 095/08(A2)11). All efforts were made to reduce the number of rats used and their suffering.

### Primary culture and hypoxia treatment of microglial cells

Twenty-five 3-day-old postnatal rats were used for the preparation of primary culture of microglia. Glial cells were isolated from the cerebrum of rat pups. Once confluent (12–14 days), microglia were isolated from the mixed glial population by a method previously described [31]. The purity of microglia was assessed by immunocytochemical labeling using OX42 (1∶100, Santa Cruz Biotechnology, Santa Cruz, CA, USA; Cat. No. sc-53086), a specific marker of microglia. Microglial cultures with >96% purity were used for the study. For immunostaining, 2.0×10^5^ cells/well were plated in poly-L-lysine coated coverslips placed in 24-well plates. For hypoxia treatment, the culture medium was changed to fresh medium for routine culture before the cells were exposed to hypoxia by placing them in a chamber filled with a gas mixture of 3% O_2_/5% CO_2_/92% N_2_ for 2, 4, 6, 12 and 24 h. To inhibit Notch signaling, microglia were pretreated with DAPT (Sigma-Aldrich, St. Louis, MO; Cat. No. D5942; DAPT dissolved in dimethyl sulfoxide) at 10 µM for 1 h and then exposed to hypoxia immediately.

### BV-2 cell culture and treatment

BV-2 cells were used for *in vitro* study because our recent studies have shown that this microglial cell line responded swiftly to hypoxic exposure [32], [33]. The culture medium was changed prior to hypoxia exposure. Hypoxia was administered by placing the cells in a chamber filled with a gas mixture of 3% O_2_/5% CO_2_/92% N_2_ for 2, 4, 6, 8 and 12 h. DAPT (10 µM) was added into the medium 1 h before hypoxia treatment.

### Double immunofluorescence labeling in cerebrum, primary culture microglia and BV-2 cells

Double immunofluorescence was carried out in postnatal rats to confirm the expression of Notch signaling in microglia as well as NF-κB activation after DAPT pretreatment. Briefly, neonatal rats were anaesthetized with 6% sodium pentobarbital administrated by intraperitoneal injection and perfused with a fixative containing 2% paraformaldehyde in 0.1 M phosphate buffer, pH 7.4. The brains were then removed and placed in the same fixative for 4 h following which they were kept at 4°C overnight in 0.1 M phosphate buffer containing 15% sucrose. Coronal postnatal brain sections of 40 µm thickness were cut using a cryostat (Leica Microsystems Nussloch GmbH, Nussloch, Germany). The sections were incubated with NICD (goat anti rabbit 1∶100, Merck KGaA, Darmstadt, Germany; Cat. No. 07-1232), Delta-1 (rabbit anti goat, 1∶50, Santa Cruz Biotechnology, Santa Cruz, CA, USA; Cat. No. sc-8155) or NF-κB (rabbit anti goat, 1∶100, Santa Cruz Biotechnology, Santa Cruz, CA, USA; Cat. No. sc-109) antibodies overnight at room temperature. After incubation, Cy3 conjugated secondary antibody was added and incubated at room temperature for 1 h. The sections were also incubated with FITC-conjugated lectin from tomato (*Lycopersicon esculentum*, 1∶100, Sigma, MO, USA; Cat. No. L-0401) and mounted using a fluorescent mounting medium with DAPI (Sigma, MO, USA, Cat. No. F6057). Cellular localization was then examined and images captured under a confocal microscope (FV1000; Olympus, Tokyo, Japan). Both primary microglial cells and BV-2 cells were fixed with 4% paraformaldehyde for 20 min and processed as described above for localization of Notch-1, Delta-1 or NICD. All the samples in different groups were processed at the same time to ensure uniform development time across all slides for suitable comparison of staining intensity against the control. All pictures were taken with the same settings for exposure and contrast and have not been digitally enhanced.

### Cell viability analysis of BV-2 and primary microglial cells

The effect of hypoxia and DAPT treatment on the viability of BV-2 and primary microglia cells was evaluated by CellTiter 96® AQueous One Solution Cell Proliferation Assay kit (Promega, WI, USA, Cat. No. G3580). 3-(4,5-dimethylthiazol-2-yl)-5-(3-carboxymethoxyphenyl)-2-(4-sulfophenyl)-2h- tetrazolium, inner salt reagent was added into each well (20 µl/well) and incubated for 4 h at 37°C in a humidified atmosphere of 5% CO_2_ and 95% air. Absorbance at 490 nm was measured using a microplate reader (GENIOS, Tecan, Switzerland). Cell viability is expressed as a percentage of control cells.

### RT-PCR

Total RNA was extracted using the RNeasy Mini kit (Qiagen, Valencia, CA, USA; Cat. No. 74104). Reverse transcription reactions were performed using the AMV Reverse Transcriptase system (Promega, Madison, Wisconsin, USA) for BV-2 cells and SuperScript® VILO™ cDNA Synthesis Kit (Invitrogen; Cat. No. 11754-050) for primary microglia. Primer pairs were designed using the primer design program (Primer 3 software version 1.0) and primer sequences for the genes and their corresponding amplicon size are listed in Table 1. 2 µl aliquot of each reverse transcription product was added to the 10 µl reaction mixture containing Fast SYBR® Green Master Mix (Invitrogen, Cat. No. 4385612) and 0.5 µM of each primer to amplify the genes in a Fast Real-Time PCR machine (Biosystems 7900HT; Life Technologies biotechnology, Germany). The expression differences for genes between the control and treated cells were calculated by normalizing with the β-actin gene expression according to the following formula: 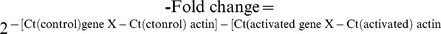. [33]

**Table 1:** Gene sequence used for RT-PCR.

Gene		Sequence
Notch-1 (rat)	Forward	ATGACTGCCCAGGAAACAAC
	Reverse	GTCCAGCCATTGACACACAC
Delta-1 (rat)	Forward	ACCATAAGCCATGCAGGAAC
	Reverse	CTTGCCATAGAAGCCAGGAG
RBP-Jκ (rat)	Forward	GAGCCATTCTCAGAGCCAAC
	Reverse	TCCCCAAGAAACCACAAAAG
Hes-1 (rat)	Forward	AGCCAACTGAAAACACCTGATT
	Reverse	GGACTTTATGATTAGCAGTGG
M-CSF (rat)	Forward	AGAGCTCCTGCCTACCAAGAC
	Reverse	TCCTAAAGGAAAGGGTCCTGA
TGF-β1 (rat)	Forward	TGCTTCAGCTCCACAGAGAA
	Reverse	TGGTTGTAGAGGGCAAGGAC
IL-10 (rat)	Forward	GAATTCCCTGGGAGAGAAGC
	Reverse	CGGGTGGTTCAATTTTTCAT
IL-6 (rat)	Forward	AGTTGCCTTCTTGGGACTGA
	Reverse	ACAGTGCATCATCGCTGTTC
TLR4 (rat)	Forward	CCAGAGCCGTTGGTGTATCT
	Reverse	TCAAGGCTTTTCCATCCAAC
MyD88 (rat)	Forward	GAGATCCGCGAGTTTGAGAC
	Reverse	CTGTTTCTGCTGGTTGCGTA
TRAF6 (rat)	Forward	GGATGCTAAGCCAGAACTGC
	Reverse	GCTACACGCCTGCATCAGTA

### Western blotting analysis

BV-2 cells were cultured and treated as described above. The cell pellets were collected and then the total proteins were extracted according to the manufacturer's instruction. Briefly, the cell pellets were collected by trypsinization. First, the cells were washed three times with 1× phosphate-buffered saline followed by 1× trypsin-EDTA (Sigma; Cat. No. T4174) to dissociate the cells. The cells were then pooled and centrifuged at a speed of 1000 rpm for 5 mins. The supernatant was then discarded and the pellets washed twice with 1× phosphate-buffered saline. Protein concentration of samples was then determined by using a protein assay kit (Bio-Rad, Hercules, CA, USA; catalog No. 500-0002). Next, the protein samples were separated on 10% sodium dodecyl sulfate-polyacrylamide gels. The proteins embedded in the gel were then transferred to polyvinylidene difluoride membranes using a semidry electrophoretic transfer cell (Bio-Rad, Hercules, CA, USA). The membranes were incubated with Notch-1, NICD, RBP-Jκ, Hes-1, TNF-α, IL-1β, NF-κB/p65, IL-10, M-CSF, TGF-β1, MyD88, TRAF6 and β-actin overnight on a shaker at 4°C. The information of the different antibodies is listed in Table 2. The membranes were incubated with horseradish peroxidase-conjugated secondary antibody (dilution 1∶10000; Sigma-Aldrich, USA) for 1 h. The proteins were detected with a chemiluminescence detection system according to the manufacturer's instruction (Supersignal West Pico Horseradish Peroxidase Detection Kit; Pierce Biotechnology, IL, USA; Cat. No. 34077) and developed on film. The band intensity was quantified using Image J software (NIH). All experiments were repeated at least in triplicate.

**Table 2:** Antibodies used for western Blotting.

Antibody	Host	Source	Dilution	Catalog number
Notch-1	Rabbit polyclonal	Santa Cruz Biotechnology, Santa Cruz, CA, USA	1∶1,000	sc-6014-R
NICD	Rabbit polyclonal	Merck KGaA, Darmstadt, Germany	1∶500	07-1232
RBP-Jκ	Rabbit polyclonal	Santa Cruz Biotechnology, Santa Cruz, CA, USA	1,200	sc-28713
Hes-1	Rabbit polyclonal	Santa Cruz Biotechnology, Santa Cruz, CA, USA	1∶1,200	sc-25392
TNF-α	Rabbit polyclonal	Chemicon, Temecula, CA, USA	1∶1,000	AB2148P
IL-1β	Rabbit polyclonal	Chemicon, Temecula, CA, USA	1∶1,000	AB1413
NF-κB/p65	Rabbit polyclonal	Santa Cruz Biotechnology, Santa Cruz, CA, USA	1∶1000	sc-109
IL-10	Rat polyclonal	Abcam, Cambridge, UK	1∶1,000	ab33471
M-CSF	Rabbit polyclonal	Santa Cruz Biotechnology, Santa Cruz, CA, USA	1∶200	sc-13103
TGF-β1	Rabbit polyclonal	Santa Cruz Biotechnology, Santa Cruz, CA, USA	1∶500	sc-146
MyD88	Rabbit polyclonal	Santa Cruz Biotechnology, Santa Cruz, CA, USA	1∶200	sc-11356
TRAF6	Mouse monoclonal	Santa Cruz Biotechnology, Santa Cruz, CA,	1∶200	sc-8409
β-actin	Mouse monoclonal	Sigma-Aldrich, MO, USA	1∶10,000	A-2228

### Nitrite concentration measurement

BV-2 cells were exposed to hypoxia for 8 hours with or without DAPT as described above and the supernatant was collected. Nitric oxide concentration was measured using a Nitric oxide colorimetric BioAssay™ Kit (US Biological, Swampscott, MA, USA; Cat. No. #K262-200) according to the manufacturer's instruction.

### Phosphorylated-NF-κB p65 protein level analysis

After Notch inhibition with DAPT, the cell pellets were collected and the nuclear proteins in control and treated BV-2 cells were extracted. Nuclear proteins were extracted according to the manufacturer's instruction in the Nuclear Extraction Kit (Chemicon, Cat. No. 2900). Briefly, the cells are disrupted using the cytoplasmic lysis buffer. Next, the cell suspension was centrifuged and the cell pellet was re-suspended in two volumes of cytoplasmic lysis buffer. Nuclear protein was extracted by adding nuclear extraction buffer to the cell lysate to separate nuclear from cytosolic proteins. Upon centrifugation, the nuclear protein was extracted in the supernatant. The protein concentration was measured by Pierce™ BCA Protein Assay Kit (Pierce Biotechnology, Rockford, USA; Cat. No. 23227). Phospho-NF-κB/p65 protein level analysis was carried out using PathScan Phospho-NF-κB/p65 (Ser536) Sandwich ELISA Kit (Cell signaling, CA, USA; Cat. No. 7173) according to the manufacturer's instruction.

### Statistical analyses

The data are presented as mean ±SD. Statistical significance of differences between control and hypoxic groups was calculated using Student's t test and differences between control, hypoxic and treatment groups was calculated using one-way analysis of variance (ANOVA). Statistical significance in control vs hypoxic microglia was represented as *p<0.05 and **p<0.01; statistical significance in control vs control+DAPT group and hypoxia vs hypoxia+ DAPT group are represented as ^#^ *p*<0.05 and ^##^ *p*<0.01.

## Results

### Notch signaling was activated in primary microglia and BV-2 cells after hypoxia

**Figure 1:**
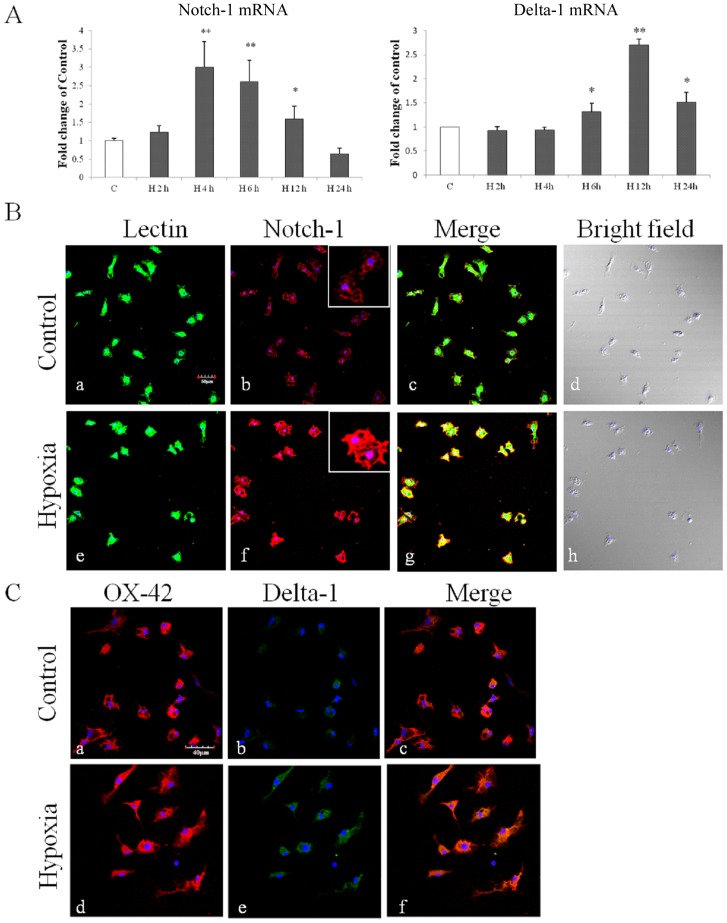
Up-regulation of Notch-1 and Delta-1 expression in primary cultured microglia following hypoxia. (**A**) Reverse transcription (RT)-PCR analysis of Notch-1 and Delta-1 mRNA expression in primary microglia exposed to hypoxia for 2, 4, 6, 12 and 24 h and control (c). Note the significant increase in Notch-1 and Delta-1 mRNA expression after hypoxia. (**B and C**) Confocal images showing Notch-1 expression (Bb, Bf; red) in primary cultured microglia labeled with lectin (Ba, Be; green) and Delta-1 expression (Cb, Ce; green) colocalized with OX-42 (Ca, Cd; red)) in both control and hypoxia for 12 h. Nuclei are stained with DAPI (blue). Note Notch-1and Delta-1 immunoflurosence intensity is markedly enhanced after hypoxia exposure (Bg, Cf) in comparison with the control (Bb, Cb). The values represent the mean ±SD in triplicate. Scale bars  = 50 µm (**B**) and 40 µm (**C**).

**Figure 2:**
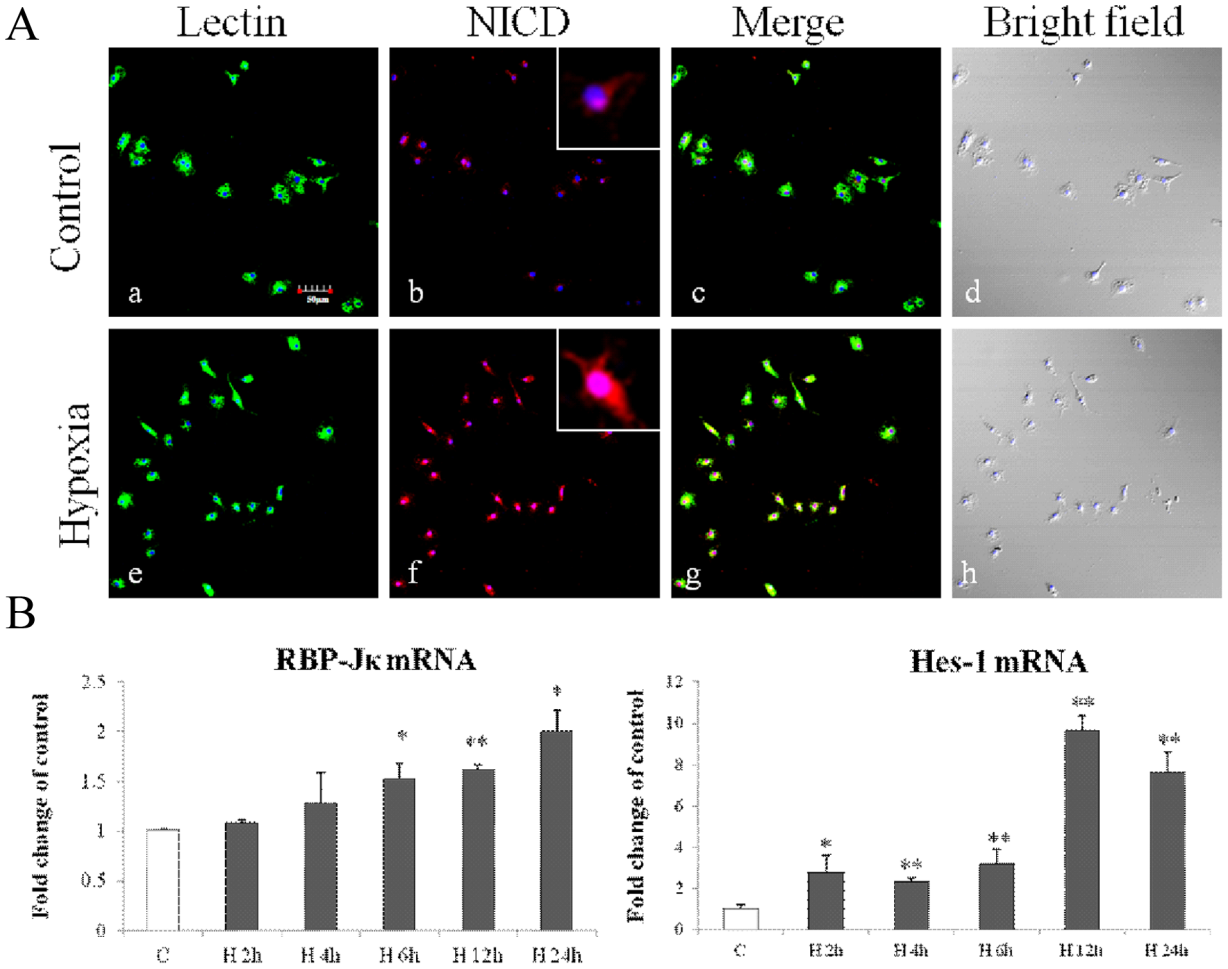
Notch signaling was activated in primary cultured microglia exposed to hypoxia. (**A**) Immunofluorescence images showing NICD expression in primary microglia labeled with lectin (a, e; green). The expression is intensely augmented both in the cytoplasm and nucleus after hypoxic treatment for 12 h (f, g) compared with the control (b, c). (**B**) Reverse transcription (RT)-PCR analysis of RBP-Jκ and Hes-1 mRNA expression in primary microglia exposed to hypoxia for 2, 4, 6, 12 and 24 h and control (c). Note the significant increase in RBP-Jκ and Hes-1 mRNA expression after hypoxia. The values represent the mean ±SD in triplicate. Significant differences between control and hypoxic BV-2 cells are expressed as *p<0.05 and ** *p*<0.01. Scale bars  = 50 µm (**A**).

**Figure 3:**
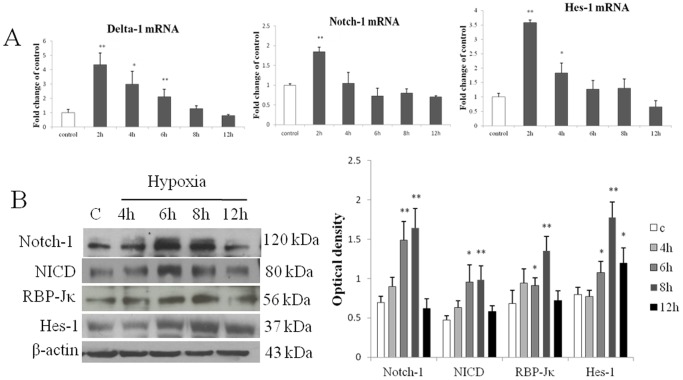
Notch signaling was expressed and activated in BV-2 cells following hypoxia. (**A**) RT-PCR analysis showing the mRNA expression of Notch-1, Delta-1 and Hes-1 mRNA in BV-2 cells exposed to hypoxia was significantly increased compared with the control. (**B**) Western blotting of Notch-1, NICD, RBP-Jκ and Hes-1 protein expression in BV-2 cells exposed to hypoxia for 4, 6, 8 and 12 h and control (c). The left panel shows specific bands of Notch-1 (120 kDa), NICD (80 kDa), RBP-Jκ (56 kDa), Hes-1 (37 kDa) and β-actin (43 kDa). The right panel is bar graphs showing significant changes in the optical density following hypoxic exposure. Note significant increase in Notch-1, NICD, RBP-Jκ and Hes-1 expression after hypoxic treatment of varying durations in BV-2 cells. Significant differences between control and hypoxic BV-2 cells are expressed as *p<0.05 and ** *p*<0.01. The values represent the mean ±SD in triplicate.

Primary microglia and BV-2 cells were subjected to hypoxia for 2–24 h and 2–12 h respectively. Notch-1 and Delta-1 mRNA expression in primary microglia was most significantly increased after hypoxia peaking at 4 h for Notch-1 and at 12 h for Delta-1 (Fig. 1A). Expression of Notch-1 and Delta-1 in primary microglia was further confirmed by immunofluorescence staining which showed that the immunofluorescence intensity of Delta-1 and Notch-1 was obviously enhanced after hypoxia (Fig. 1B and C). Notch signaling activation in primary microglia after hypoxia was confirmed by the detection of enhanced immunofluorescence intensity of NICD both in the cytoplasm and nucleus (Fig. 2A). RBP-Jκ mRNA expression was progressively increased in primary microglia at various time points after hypoxia (Fig. 2B). As the main target gene of Notch signaling, Hes-1 mRNA expression was concurrently increased at different time points after hypoxia, peaking at 12 h in which the increase was more than 9 folds compared with the control in primary microglia (Fig. 2B). The expression and activation of Notch signaling was also observed in BV-2 cells (Fig. 3). Delta-1 and Notch-1 mRNA expression was elevated being most significantly at 2 h after hypoxia (Fig. 3A). Western blot analysis in BV-2 cells also showed that Notch-1 protein expression was progressively increased after hypoxic exposure (Fig. 3B). NICD protein expression was increased especially at 6–8 h after hypoxia (Fig. 3B), and protein expression of RBP-Jκ also showed a significant increase being most pronounced at 8 h (Fig. 3B). Increase in Hes-1 mRNA and protein expression after hypoxia was corroborated in hypoxic BV-2 cells (Fig. 3A and B).

### DAPT treatment inhibited Notch signaling activation in hypoxic microglia

**Figure 4:**
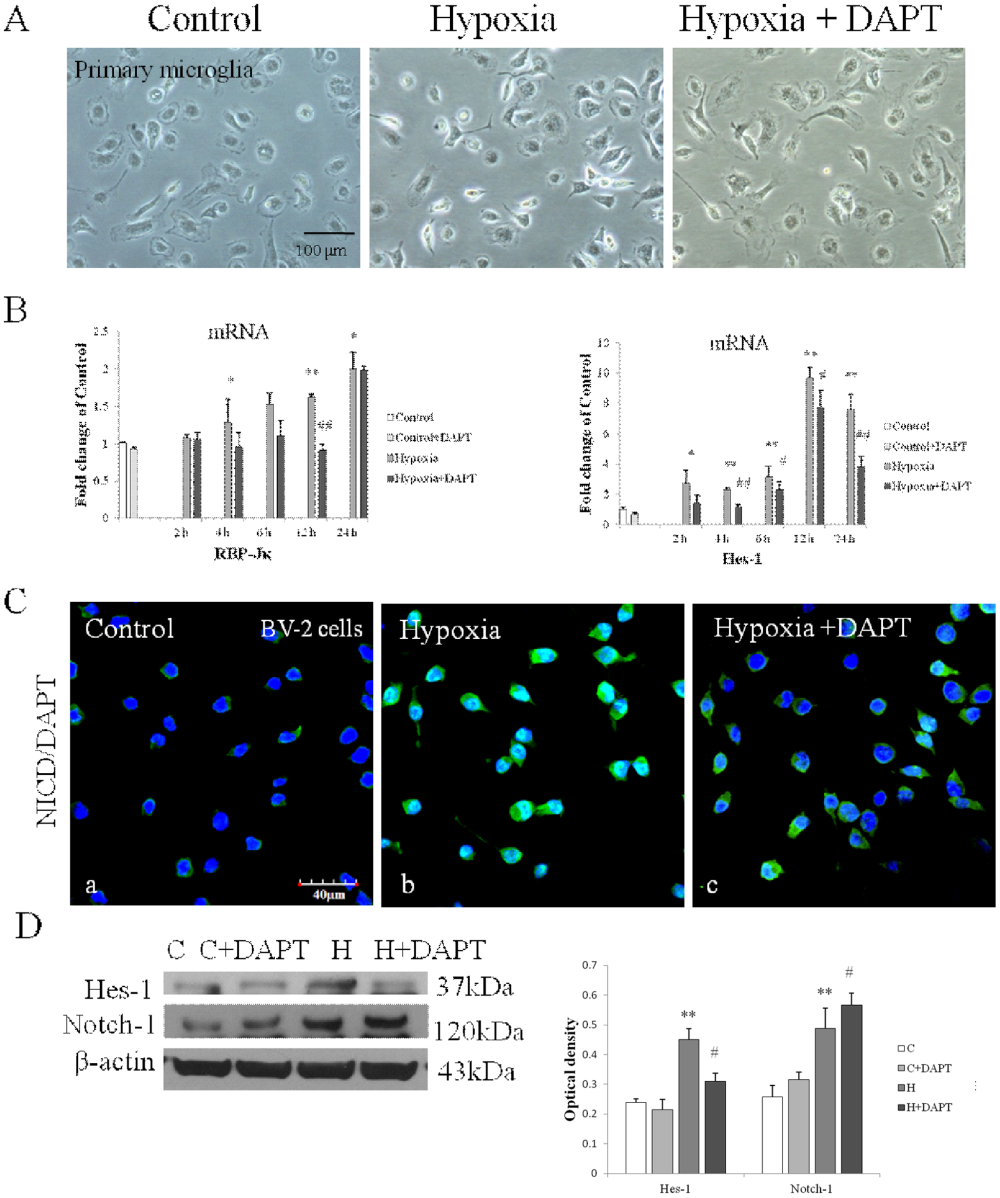
Notch signaling blockade in primary microglia and BV-2 cells by DAPT. (**A**) No obvious morphological difference was observed in Hypoxia and Hypoxia+DAPT groups compared with the control primary microglia under the phase-contrast microscope. (**B**) The mRNA expression of RBP-Jκ and Hes-1 in primary microglia was significantly decreased in Hypoxia+DAPT group compared with Hypoxia group shown by RT-PCR analysis. (**C**) Confocal images showing NICD expression in BV-2 cells of different groups. NICD immunofluorescence intensity was reduced both in cytoplasm and nucleus in Hypoxia +DAPT BV-2 cells (Cc) compared with hypoxic BV-2 cells (Cb). (**D**) Western blotting of Notch-1 and Hes-1 protein expression in BV-2 cells after DAPT pretreatment. The left panel shows specific bands of Notch-1 (120 kDa), Hes-1 (37 kDa) and β-actin (43 kDa). The right panel is bar graphs showing Notch-1 protein expression was increased in Hypoxia+DAPT group compared with hypoxic BV-2 cells; while increase in Hes-1 protein expression after hypoxia was significantly inhibited in DAPT pretreated hypoxic BV-2 cells. Significant difference between control vs hypoxia groups is shown as *p<0.05 and **p<0.01; Significant difference between hypoxia vs hypoxia+DAPT groups is shown as ^#^ *p*<0.05 and ^##^ *p*<0.01. The values represent the mean ±SD in triplicate. Scale bar in C = 40 µm.

DAPT was used to investigate the effect of Notch activation in microglial response. Notch inhibition by DAPT treatment was first confirmed both in primary microglia and BV-2 cells. There was no change in cell density and cell morphology as observed in primary microglia (Fig. 4A) and BV-2 cells (data not shown) after hypoxia with or without DAPT pretreatment. No cytotoxic effect of DAPT was observed as investigated by 3-(4,5-dimethylthiazol-2-yl)-5-(3-carboxymethoxyphenyl)-2-(4-sulfophenyl)-2h- tetrazolium, inner salt (data not shown). Both RBP-Jκ and Hes-1 mRNA expressions were significantly inhibited in DAPT pretreated primary microglia after different durations of hypoxia (Fig. 4B). In BV-2 cells, immunofluorescence staining showed a decrease in NICD immunofluorescence and nuclear translocation in Hypoxia +DAPT group compared with the Hypoxia group (Fig. 4C). The decrease in Hes-1 protein expression was also observed in Hypoxia +DAPT group (Fig. 4D). It is noteworthy that Notch-1 protein expression was increased significantly in DAPT pretreated hypoxic BV-2 cells compared with cells subjected to hypoxia exposure with DAPT treatment (Fig. 4D).

### Notch signaling blockade in microglia inhibited production of inflammatory mediators

**Figure 5:**
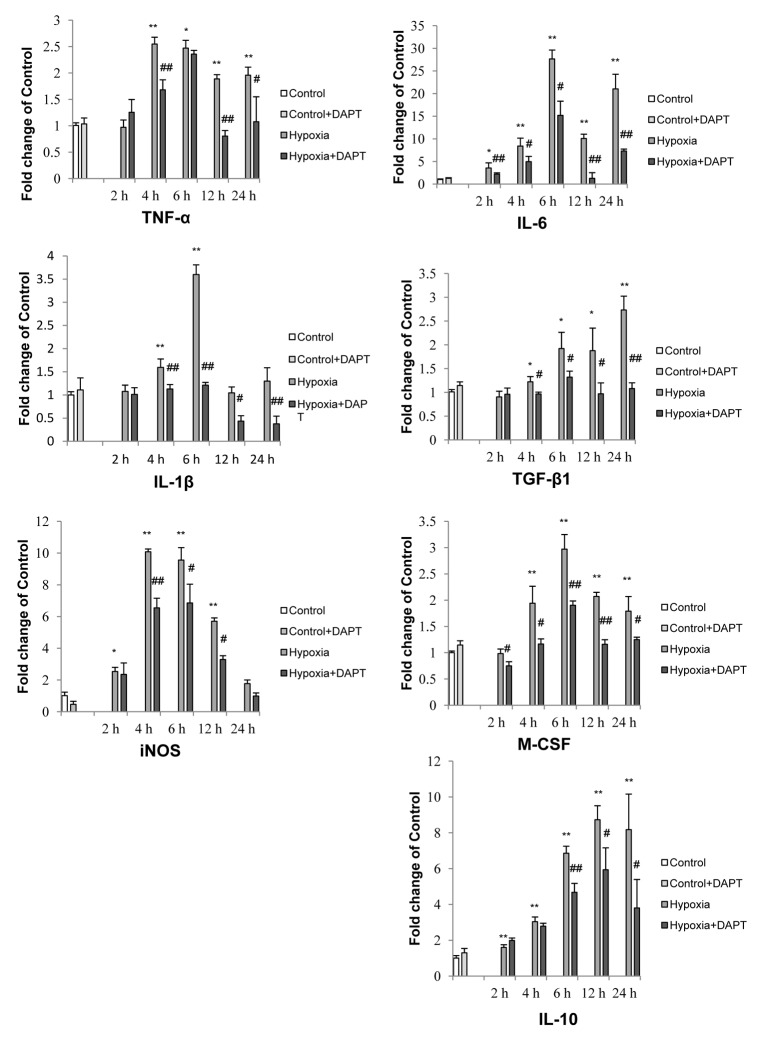
Notch blockade altered the mRNA expression of inflammatory cytokines and iNOS induced by hypoxic stress in primary microglia. Reverse transcriptase (RT)-PCR analysis of *TNF-α*, *IL-1β*, *iNOS, TGF-β1, M-CSF, IL-10 and IL-6* gene expression in primary microglia exposed to different duration of hypoxia with or without DAPT pretreatment. Note that mRNA expression of all the above mentioned genes is increased significantly to varying extents after hypoxic exposure for different duration. Significant difference between control vs hypoxia groups is shown as *p<0.05 and **p<0.01; significant difference between hypoxia vs hypoxia+DAPT groups is shown as ^#^ *p*<0.05 and ^##^ *p*<0.01. The values represent the mean ±SD in triplicate.

**Figure 6:**
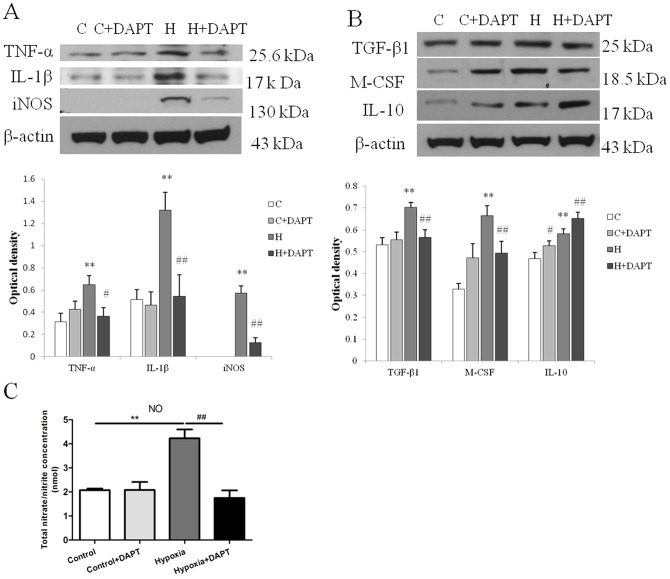
Notch blockade altered protein expression of inflammatory cytokines, iNOS and nitric oxide (NO) secretion in hypoxic BV-2 cells. (A and B) Western blotting of TNF-α, IL-1β and iNOS (A); TGF-β1, M-CSF and IL-10 (B) protein expression in BV-2cells following 8 h of hypoxic exposure and DAPT pretreatment. The upper panel shows specific bands of TNF-α (25.6 k Da), IL-1β (17 kDa), iNOS (130 kDa) and β-actin (43 kDa) (A); TGF-β1 (25 kDa), M-CSF (18.5 kDa), IL-10 (17 kDa) and β-actin (43 kDa) (B). The lower panel in A and B are bar graphs showing significant changes in the optical density in protein expression of different groups. Note the decrease in TNF-α, IL-1β and iNOS expression (A) as well as TGF-β1 and M-CSF expression (B) in hypoxia+DAPT group compared with hypoxic BV-2 cells. A noteworthy feature was the increase in IL-10 protein expression after DAPT pretreatment in hypoxic BV-2 cells (B). (**C**) NO production in supernatant of different groups of cells. Note the NO production, which is increased after hypoxic exposure for 8 h is decreased nearly to basal level after hypoxic exposure in the DAPT treated BV-2 cells. Significant difference between control vs hypoxia groups is shown as *p<0.05 and **p<0.01; significant difference between control vs hypoxia and hypoxia vs hypoxia+DAPT groups is shown as ^#^ *p*<0.05 and ^##^ *p*<0.01. The values represent the mean ±SD in triplicate.

As an increase in expression of inflammatory mediators is considered the hallmark feature of activated microglia, we next investigated whether Notch inhibition would affect the expression and secretion of inflammatory mediators by hypoxic microglia. It was found that hypoxia resulted in a significant increase in mRNA expression of TNF-α, IL-1β and iNOS in primary microglia which was partially inhibited following Notch signaling blockade (Fig. 5). Similarly, western blot results showed a significant decrease in TNF-α, IL-1β and iNOS protein expression levels in hypoxic BV-2 cells pretreated with DAPT (Fig. 6A).

We next investigated the expression of other inflammatory mediators, including M-CSF, IL-6, IL-10 and TGF-β1 in hypoxic primary microglia. Notch blockade showed a universal inhibition of the mRNA expression of M-CSF, IL-6, TGF-β1 and IL-10 (Fig. 5). In parallel to the decrease in mRNA expression with Notch blockade, DAPT pretreatment also inhibited M-CSF and TGF-β1 protein expression in BV-2 cells across different groups with the exception of IL-10 whose expression was increased with DAPT pretreatment in BV-2 cells of control and after hypoxia for 8 h (Fig. 6B). In addition, the increase in NO after hypoxia was significantly reduced with DAPT treatment in hypoxic BV-2 microglia (Fig. 6C).

### DAPT blockade of Notch signaling in hypoxic microglia decreased NF-κB pathway activation

**Figure 7:**
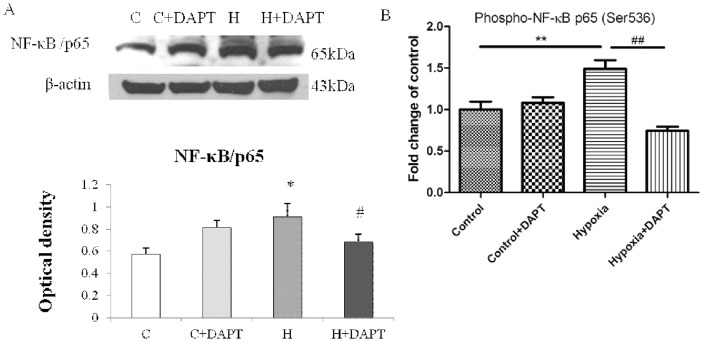
DAPT treatment inhibited NF-κB activation and translocation induced by hypoxic stress in BV-2 cells. (**A**). Western blot analysis of NF-κB/p65 protein expression in BV-2 cells of different groups. The upper panel shows specific bands of NF-κB/p65 (65 kDa) and β-actin (43 kDa) and the lower panel bar graph showing significant changes in the optical density of different groups. Note the NF-κB/p65 protein expression, which is increased after hypoxic exposure in control BV-2 cells, is significantly decreased after hypoxic exposure in DAPT treated BV-2 cells. (**B**) ELISA analysis of phospho-NF-kB/p65 in nucleus of different groups of BV-2 cells showing the content of phospho-NF-kB/p65 in nucleus is increased in BV-2 cells after hypoxic stress; however, phospho-NF-kB/p65 content is drastically reduced in hypoxic BV-2 cells pretreated with DAPT compared with the hypoxic BV-2 cells. Significant difference between control vs hypoxia groups is shown as *p<0.05 and **p<0.01; significant difference between hypoxia vs hypoxia+DAPT groups is shown as ^#^ *p*<0.05 and ^##^ *p*<0.01. The values represent the mean ±SD in triplicate.

We have reported previously that Notch-1 signaling could transactivate NF-κB/p65 as evidenced by the fact that NF-κB/p65 DNA binding ability was inhibited after Notch inhibition in LPS-activated microglia [34]. As NF-κB is an essential transcription factor for cytokines and iNOS expression in microglia, we investigated whether NF-κB pathway would be affected by Notch signaling in hypoxic microglia. Western blotting analysis indicated a significant increase in NF-κB/p65 in BV-2 cells exposed to hypoxia, but the increase was significantly prevented when the cells were pretreated with DAPT and exposed to hypoxia (Fig. 7A). ELISA analysis showed that phospho-NF-κB/p65 protein expression in nucleus was increased by 1.5 fold following hypoxia, but the increase was inhibited in hypoxic BV-2 cells pretreated with DAPT (Fig. 7B).

### Notch blockade inhibited TLR4-Myd88-TAFR6 pathway that contributed to deactivation of NF-κB pathway in hypoxic microglia

**Figure 8:**
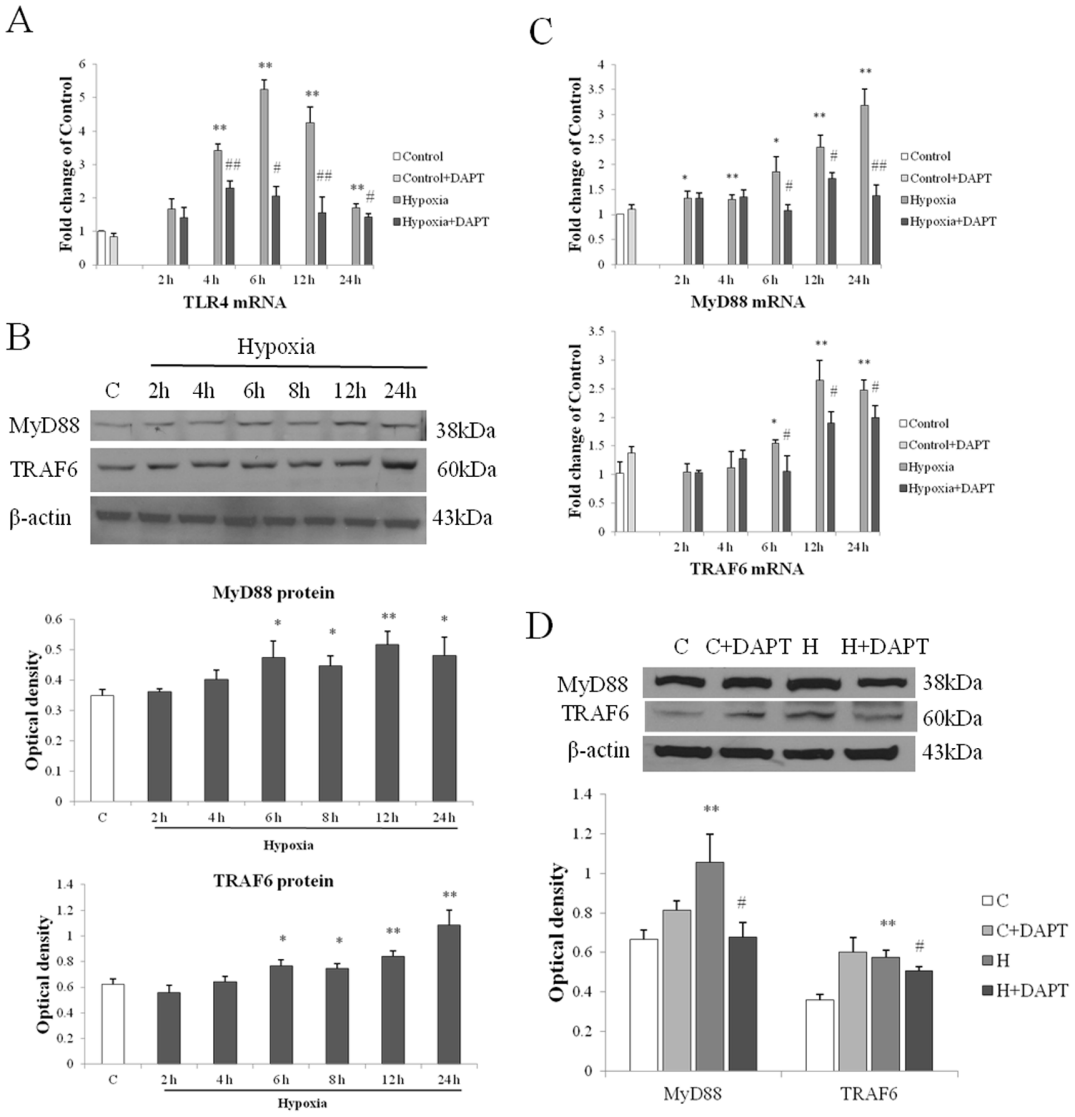
TLR4/MyD88/TRAF6 pathway was inhibited in hypoxic microglia with Notch signaling blockade. **(A)** Notch blockade suppressed TLR4 mRNA expression. RT-PCR analysis showing the hypoxia induced increase in mRNA expression of TLR4 in primary microglia was significantly suppressed when pretreated with DAPT. (**B**) Western blotting of MyD88 and TRAF6 protein expression in BV-2 cells exposed to hypoxia for 2, 4, 6, 8, 12 and 24 h and control (c). The upper panel shows specific bands of MyD88 (38 k Da), TRAF6 (60 k Da) and β-actin (43 kDa). The lower panel is bar graphs showing significant changes in the optical density following hypoxic exposure. Note the MyD88 and TRAF6 protein expression after hypoxia is significantly increased. (**C**) RT-PCR analysis showing the hypoxia induced increase in mRNA expression of MyD88 and TRAF6 in primary microglia is significantly suppressed when pretreated with DAPT. (**D**) Western blotting of MyD88 and TRAF6 protein expression in BV-2 cells exposed to hypoxia for 8 h, hypoxic BV-2 cells pretreated with DAPT and corresponding control (c). The bar graphs showing increase in MyD88 and TRAF6 protein expression induced by hypoxia is significantly suppressed in DAPT pretreated group. Significant difference between control vs hypoxia groups is shown as *p<0.05 and **p<0.01; significant difference between hypoxia vs hypoxia+DAPT groups is shown as ^#^ *p*<0.05 and ^##^ *p*<0.01. The values represent the mean ±SD in triplicate.

As NF-κB phosphorylation and translocation induced by hypoxia was hindered by Notch inhibition, we next investigated whether this is due to an interference with upstream NF-κB signaling pathway via Toll like receptor 4 (TLR4) signaling through Myd88 and TRAF6. Activation of NF-κB signaling pathway in microglia has been reported to be mediated by many factors, the best recognized and characterized for this being the TLR4 after stimulation by its potent ligand LPS [35]–[37]. We previously reported that an increase in TLR4 expression can also mediate NF-κB signaling pathway activation in microglia after hypoxic exposure [33]. Activation of TLR4 has been reported to trigger a cascade of cellular signals that culminate in the activation of NF-κB which leads to inflammatory gene expression. Therefore, we investigated whether Notch signaling can interfere in the NF-κB activation via the TLR4-NF-κB pathway. Recent evidence also supports our hypothesis by suggesting that there exists an intricately linked crosstalk between Notch and Toll like receptor signaling pathways [15], [17], [38]–[40]. In this study, we found a significant inhibition of TLR4 mRNA expression in hypoxic primary microglia pretreated with DAPT (Fig. 8 A).

TLR4 signaling activation in microglia after LPS stimulation triggers recruitment of the adaptor molecules, predominantly myeloid differentiation primary response 88 (MyD88) [41], followed by interleukin-1 receptor-associated kinase and TNFR-associated factors (TRAF6). TRAF6 activates IkappaB kinase leading to the degradation of IκB, which frees NF-κB to translocate to the nucleus, where it binds to κB sites in the promoter region of genes encoding proinflammatory cytokines [42], [43]. By western blot analysis, we found a significant increase in MyD88 and TRAF6 protein expression after different durations of hypoxia (Fig. 8B). This suggests that the MyD88 dependent pathway was activated in microglia after hypoxia exposure.

We next sought to determine whether DAPT blockade of Notch signaling would inhibit the expression of MyD88 and TRAF6. In primary microglia, a significant increase in both MyD88 and TRAF6 mRNA expression was observed after varying hypoxia exposure. In Hypoxia+ DAPT group, MyD88 and TRAF6 expression was substantially suppressed when compared with cells treated by hypoxia alone (Fig. 8C). DAPT inhibition of MyD88 and TRAF6 protein expression was also found in BV-2 cells after hypoxic exposure (Fig. 8D).

### NICD expression in cerebral microglia after hypoxic exposure in postnatal rats

**Figure 9:**
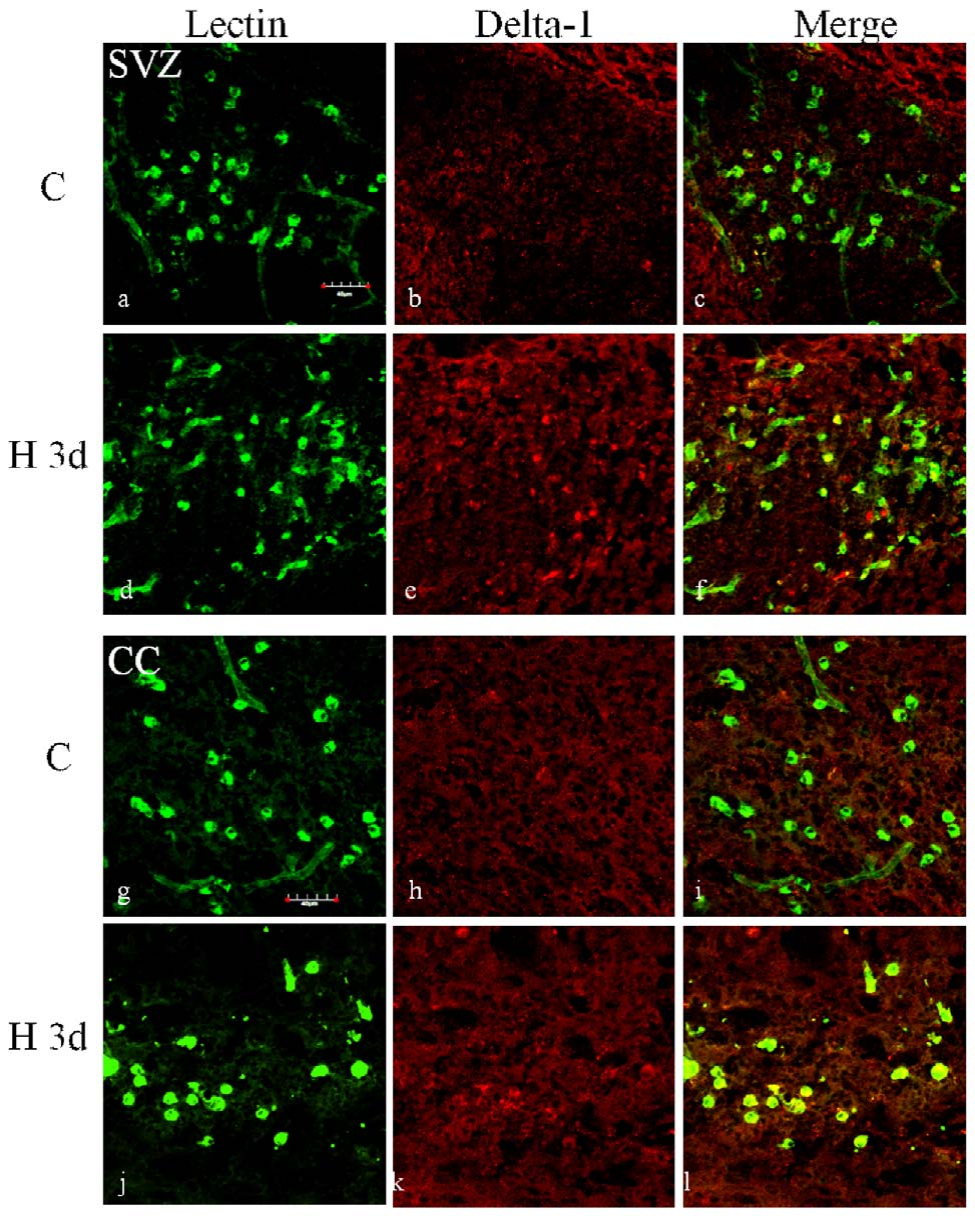
Delta-1 expression was increased in the microglial cells in subventricular zone and corpus callosum of neonatal rats following hypoxic exposure. Confocal images showing the distribution of lectin (green) and Delta-1 (red) immunoreactive microglial cells in the subventricular zone (a–f) and corpus callosum (g–l) of neonatal rats at 3 days after hypoxic exposure and the corresponding control. Very weak Delta-1 expression (arrows) is detected in the SVZ of control rats, but the immunoflurorescence intensity is enhanced and more Delta-1 positive microglial cells are observed after hypoxia. In the corpus callosum, Delta-1 expression is barely detected in microglia of control rats (h and i) and some Delta-1 positive cells colocalized with lectin (arrowheads) are seen after hypoxia (k and l). Scale bar  = 40 µm.

**Figure 10:**
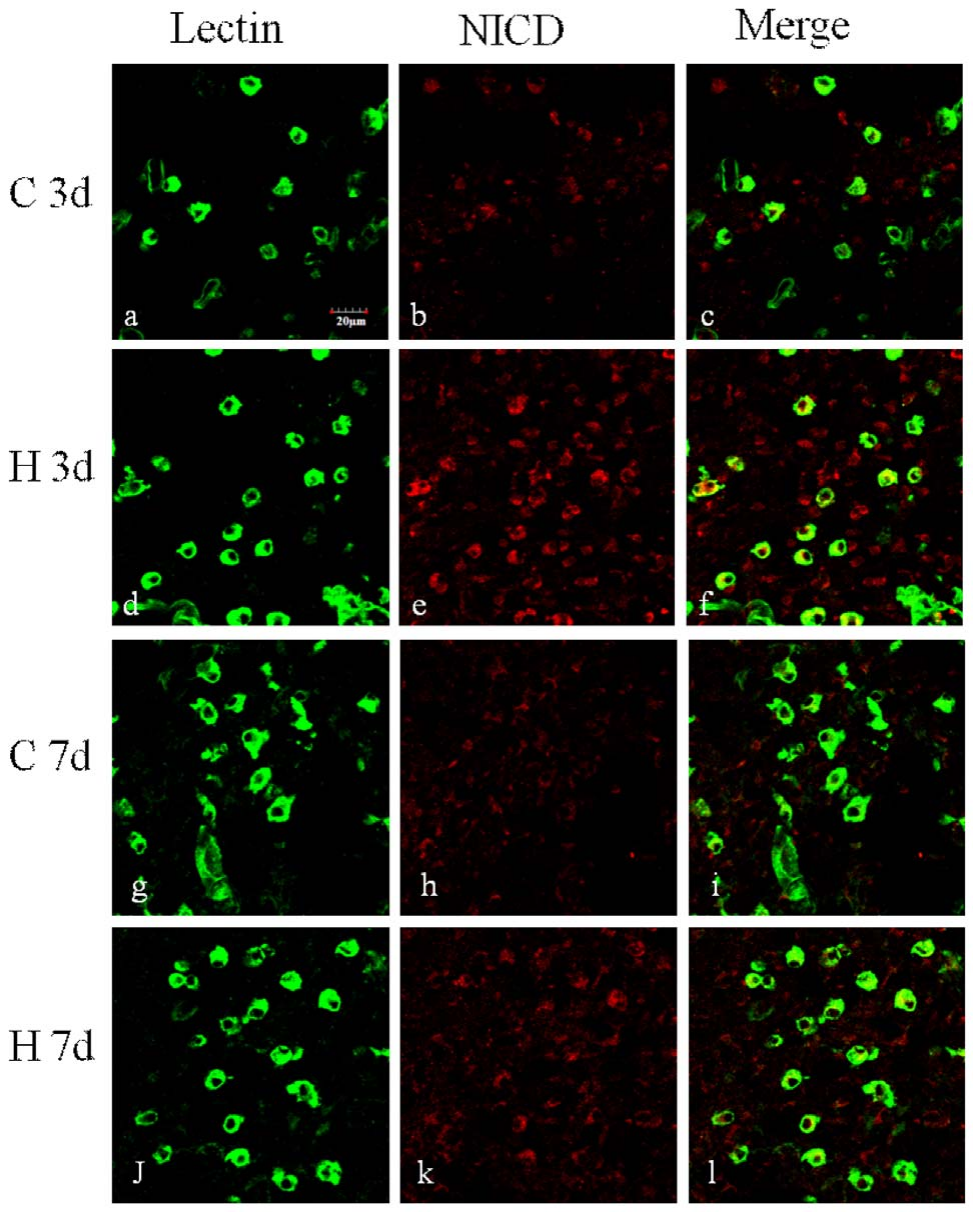
NICD expression was increased in the corpus callosum of neonatal rats following hypoxic exposure. Confocal images showing the expression of NICD (red) in the corpus callosum of neonatal rats 3 and 7 days after hypoxia and the corresponding control. Microglial cells were labeled with lectin (green). Very week NICD immunofluorescence intensity was observed in lectin-positive microglia in the control rats of both 3 (b–c) and 7 (g–i) days. NICD immunofluorescence intensity in microglia is enhanced after hypoxic exposure at 3 (d–f) and 7 (j–l) days after hypoxia, especially at 3 days (d–f) in comparison with the control (j–l)). Nuclei are stained with DAPI (blue). Scale bars  = 20 µm.

Arising from the *in vitro* results showing the roles of Notch signaling in microglia activation, we then extended our investigation to determine whether Notch signaling may play a role in microglia mediated inflammation in *vivo*. In the developing brain after hypoxic injury, Delta-1 immunoexpression was markedly increased on microglia in the corpus callosum (CC) and subventricular zone (SVZ) (Fig. 9). To assess the activation of Notch signaling in microglia in the developing brain following a hypoxic injury, we further profiled the change of NICD expression in microglia in the CC. *In vivo*, NICD was noticeably increased in lectin-labeled microglia at 3 d and 7 d after hypoxia compared with the control (Fig. 10).

### DAPT pretreatment inhibited NF-κB activation in the microglia of postnatal rats subjected to hypoxia

**Figure 11:**
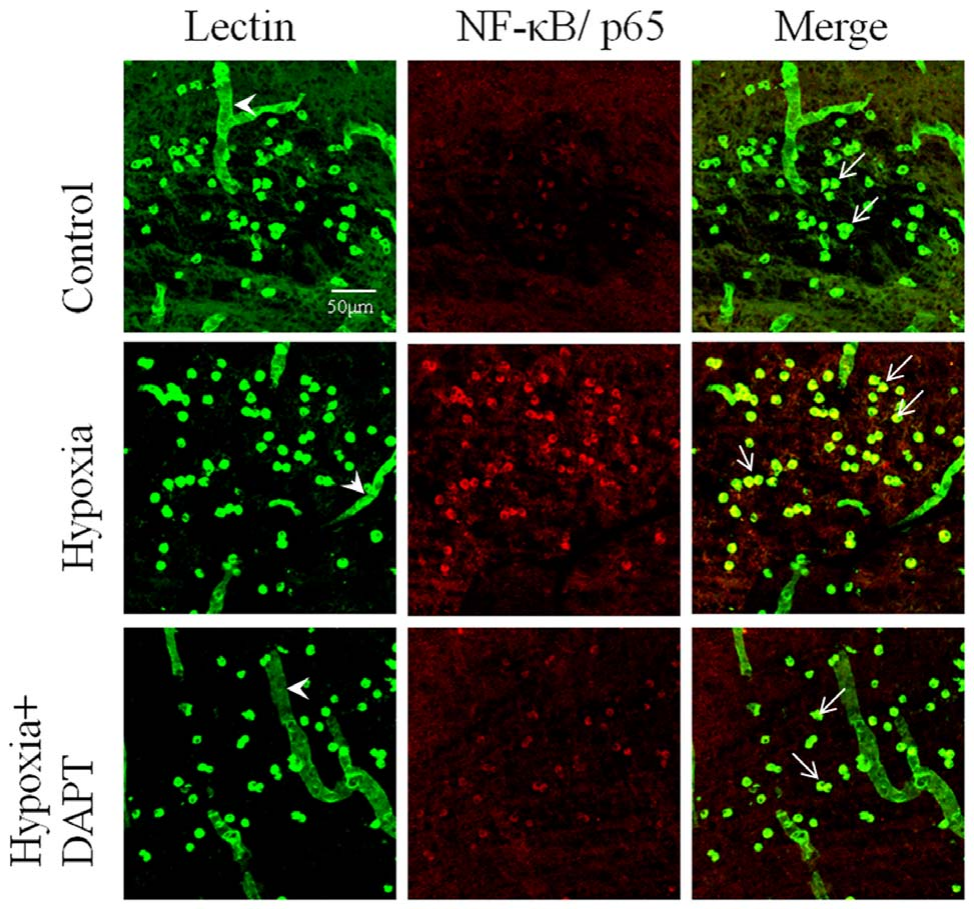
DAPT pretreatment inhibited the increase in NF-κB immunoexpression in microglia of neonatal rats after hypoxic treatment. Confocal images showing the expression of NF-κB in lectin-labeled (green) microglia (arrows) in the corpus callosum of control (a–c), hypoxia (d–f) and hypoxia +DAPT (g–i) rats at 24 h after hypoxic exposure. Increase in NF-κB expression in microglia of the corpus callosum was evident in hypoxic rats (e,f). In hypoxia +DAPT rats, increase in NF-κB was inhibited when compared with that in the hypoxic rats (h,i). Note lack of NF-κB expression in lectin positive blood vessels (arrowhead). Scale bar  = 20 µm.

In postnatal rats subjected to hypoxia, NF-κB immunofluorescence was markedly enhanced in hypoxic microglia cells when compared to cells in normal control rats. In rats given DAPT pretreatment, hypoxia-induced upregulation of NF-κB expression was noticeably reduced (Fig. 11).

## Discussion

Notch signaling expression and activation has been reported in a variety of cells and in different diseases yet its expression and function in microglia have remained elusive. Notch-1 signaling is most widely studied in immune cells including macrophages and microglia [20], [21], [39], [44], [45]. Recent studies by us have demonstrated the presence of Notch-1 signaling especially in activated microglia. We have shown that Notch signaling mediates inflammatory cytokine production in microglia challenged by LPS [20], [34]. As hypoxia is a common factor in many neuroinflammatory disorders, we sought to investigate the putative mechanism of Notch in hypoxia induced neuroinflammation in microglia. Here we provide evidence of a novel role for Notch signaling in regulating microglia activation in neuroinflammation which is linked to hypoxia. A major finding is the activation of canonical Notch signaling that regulates microglia activation after hypoxic exposure both *in vitro* and *in vivo*. Additionally, we have shown that Notch signaling-induced microglia activation is partially mediated by NF-κB via TLR4-MyD88-TRAF6 signaling.

The present results show that Delta-1 expression was increased in both primary microglia and BV-2 cells after hypoxia which differs from the decreased Delta-1 expression in LPS-stimulated BV2 cells [20]. The observed increase in Delta-1 expression was also replicated *in vivo* as reflected by the enhanced immunofluorescence intensity of Delta-1 in the SVZ and CC of postnatal rats following hypoxic exposure. Furthermore, activation of Notch-1 signaling was confirmed by the increase in NICD expression and an increase in expression of RBP-Jκ, which works together to initiate the downstream pathway. Additionally, there was also a significant increase in Hes-1, the main target gene of Notch signaling in microglia after hypoxia. This is especially evident in the primary cultures of microglia in which Hes-1 increase was about 9 folds. This suggests the involvement of Hes-1 in microglia response after hypoxic exposure although the specific mechanism for this remains to be elucidated.

Notch signaling in various cell types has been reported to be activated under hypoxic conditions *in vitro* and *in vivo* in models of pathological conditions such as leukemia and cancer. In our study, we demonstrated the upregulation of Notch, Delta and RBP-Jk after hypoxia in BV-2 microglia cells. The mechanism via which hypoxia induces Notch signaling remains unclear although there have been suggested mechanisms, and whether these mechanisms are conserved across different cell types. For example, the upregulation of hypoxia-inducible factors (HIF) has been implicated in hypoxia-induced Notch signaling [46] which can be suppressed with the use of HIF inhibitor treatment [47]. Hypoxia may also activate Notch signaling by upregulating the expression of the Notch ligand Delta-like 4 in a positive feedback manner and also function to upregulate proteins that are dependent on Notch-signaling for a synergistic effect [48]. It is noteworthy that expression of both Notch receptor Notch-1 and ligand Delta-1 on microglia is increased after hypoxia suggesting that the Delta-1 ligands secreted may act through an autocrine as well as paracrine manner on the Notch receptors in view of the close proximity of microglial cells, which often exist in cell clusters. In neural stem cells, Notch signaling is activated on direct cell-to-cell contact as a result of interactions between Notch receptors and their ligands to regulate neural stem cell proliferation and differentiation. The expression of Notch receptors on microglia surrounding neural progenitor cells suggests that Notch ligands may act via a paracrine manner between microglia and neural stem cells. Furthermore, microglia is also capable of carrying out juxtacrine Notch signaling through direct cell-cell communication between Notch receptors of adjacent cells [49]. The binding between neighboring cells has been reported to assist in augmenting the receptor and ligand production, resulting in spatial patterning of longer range patterns through a positive feedback mechanism [50], [51]. This may prove beneficial in generating the observed coordinated increases in ligand, receptor and binding targets in our study in response to hypoxia.

Besides microglia, a few Delta-1-positive lectin-negative cells were also observed in the corpus callosum of neonatal rats. The identity of these cells remains unclear. However, as they were distributed in the white matter in which immature glial cells are known to preponderate, the upregulation and concomitant release of Delta-1 could function to promote Notch signaling in early oligodendrocytes and astrocytes. Notch signaling has been reported to play roles in oligodendrocyte precursor differentiation and negatively regulate neurogenesis through endolysosomal degradation in astrocytes [52] [53] [54]. Most commonly, Notch signaling is implicated in neural progenitor cells to regulate the transition between proliferation and neurogenesis [55].

To further ascertain the functions of Notch signaling in microglia response after hypoxia, we applied a γ-secretase inhibitor, namely DAPT which impaired NICD synthesis to block Notch signaling activation. Hes1 upregulation induced by hypoxia was inhibited in DAPT pretreated cells and the inhibition of γ-secretase activity by DAPT also resulted in the decrease in RBP-Jk mRNA expression, possibly through the effect of hypoxia-induced upregulation of Notch signaling. It is striking that blockade of Notch resulted in an almost universal inhibition of expression and production of several cytokines with the exception of IL-10. IL-10, which is generally considered as an anti-inflammatory factor was increased after DAPT treatment. DAPT inhibited IL-10 mRNA expression beginning at 4 h after hypoxia; however western blot analysis in BV-2 cells showed that DAPT increased IL-10 protein expression after 8 h of hypoxic exposure. IL-10 is generally considered as an anti-inflammatory factor during inflammation. Here we showed that IL-10 expression was suppressed by Notch signaling in microglia after hypoxic exposure. This observation suggests that Notch signaling activation not only induces the expression of pro-inflammatory factors, but also inhibits the expression and secretion of some anti-inflammatory factors. In addition, IL10 was reported to inhibit microglia production of TNF-α, IL-1β, NO, ROS and suppresses NF-κB activation [56]; therefore, the increase in IL-10 after Notch signaling inhibition may also contribute to the inhibition of NF-κB activation. However, the exact regulating mechanism of Notch signaling to IL-10 is obscure. It has been reported IL10 expression was mediated by MAPK and Akt pathway [57]; however, whether Notch signaling acts directly on IL10 or through MAPK and Akt pathway remains to be investigated. Another feature worthy of note is the effect of Notch signaling on TGF-β1 expression in hypoxic microglia. A possible cross talk between Notch signaling and TGF-β1 pathway has been reported in adenocarcinomic human alveolar basal epithelial cells and rat hepatic stellate cells [29], [58]; however, such crosstalk in microglia has not been reported and needs further investigation.

NF-κB is a transcription factor known to regulate genes of a spectrum of processes including inflammation. The canonical pathway is induced by most physiological NF-κB stimuli including signals emanating from cytokine receptors for example, TLR4. The canonical pathway mainly leads to phosphorylation of IκBα and nuclear translocation of mostly p65-containing heterodimers [59]. From the structure and the activated process of NF-κB pathway, it is not surprising that NF-κB activity is tightly controlled at multiple levels by positive and negative regulatory elements. Accumulating evidence supports the existence of important but poorly understood cross-talk between Notch and NF-κB pathway in many cells, including macrophage and microglia [15], [34], [59], [60]. In our previous study we have also demonstrated that Notch blockade can inhibit NF-κB gene binding activity in microglia after stimulation with LPS [34]. We show here that Notch blockade can inhibit NF-κB/p65 expression and translocation into the nucleus induced by hypoxia suggesting that Notch pathway enhances the release of NF-κB dimers that include NF-κB/p65. This has led us to hypothesize that some elements or factors which function in the release and translocation of NF-κB/p65 might have been affected after Notch signaling by DAPT. This notion is further supported by the significant decrease in TLR4, MyD88 and TRAF6 mRNA as well as MyD88 and TRAF6 protein expression after Notch inhibition in microglia following hypoxic exposure. This suggests that Notch signaling may mediate hypoxia induced TLR4 expression which subsequently activates the MyD88 and TRAF6 expression. Hence, Notch signaling blockade may act directly on MyD88 or TRAF6 as suggested in a study investigating Notch-TLR in macrophages [15]. The difference in Notch blockade may be due to the use of varying cell models and methodology. Nonetheless, the present results have shown that inhibition of Notch signaling may exert its influence through TRAF6 on NF-κB. However, as NF-κB activity is controlled at different levels by positive and negative regulatory elements, multiple targets may exist for the action of Notch signaling in NF-κB activity. In addition, HIF-1α has been reported to mediate TLR4-NF-κB expression in hypoxic microglia and interaction between HIF-1α and Notch signaling has been reported in many cell types [61], [62]. It was reported in human embryonic kidney 293T cells that NICD enhances recruitment of HIF-1α to its target promoters and depresses HIF-1α function by sequestering factor-inhibiting HIF-1α away from HIF-1α after hypoxia stress [62]. Therefore, we speculate that Notch signalling blockade by DAPT may also repress HIF-1α activity, thereby inhibiting the expression of downstream molecular signaling. Nevertheless, this hypothesis requires further investigation.

DAPT is a γ-secretase inhibitor, which is a powerful blocker of Notch activity. Hence, the effect of DAPT inhibition e.g. on inflammation may be inferred as the effect of interfering with Notch intracellular part NICD synthesis. On the other hand, although γ-secretase inhibitors may be a useful in screening for involvement of the Notch-signaling pathway, genetic approaches such as knockdown or over expression studies are necessary for more definitive conclusions regarding such involvement.

The present results derived from primary microglia and BV-2 cells subjected to hypoxic exposure *in vitro* have prompted us to extend our investigation to examine the expression and function of Notch signaling in activated microglia in a hypoxia animal model. The most striking feature was the activation of Notch signaling in the developing brain after hypoxic injury. Activation of Notch signaling in microglia of postnatal rats after hypoxia was followed by an increase in NICD expression in amoeboid microglial cells localized in the CC. The function of Notch signaling activation was confirmed by the fact that DAPT pretreatment significantly prevented NF-κB activation in microglia of postnatal rats after hypoxia exposure. Our findings are consistent with the literature that Notch-1 antisense mice exhibited significantly lower numbers of activated microglia and reduced proinflammatory cytokine expression in the ipsilateral ischemic cortices compared to nontransgenic mice. Microglial activation was also attenuated in Notch-1 antisense cultures and in nontransgenic cultures treated with γ-secretase inhibitor, which blocks the proteolytic cleavage and activation of Notch [21]. Some studies, however, have reported an opposing role of Notch signaling pathway in the activation of microglia and in the control of inflammatory reactions in the CNS [22]. Notwithstanding, it is unequivocal from the present results as well as from others that Notch receptor and its ligands are constitutively expressed by microglia and that Notch signaling pathway is activated after hypoxia and is functional in regulating NF-κB during inflammatory response.

To summarize, this study has demonstrated the increase of Notch signaling in activated microglia. As microglia-mediated brain inflammation is a hallmark feature of neurodegenerative diseases and is a prominent sequel of many acute forms of brain injury, anti-inflammatory treatment may act to reduce neurodegeneration and brain injury. Our finding that Notch signaling can promote microglia activation presents a potential molecular target for the development of CNS anti-inflammatory drugs. However, considering that Notch signaling is expressed on a variety of cells including stem cells in the CNS, the use of Notch signaling inhibitors such as DAPT as a potential therapeutic agent in CNS disorders awaits further consideration.

## References

[1] Gazave E, Lapebie P, Richards GS, Brunet F, Ereskovsky AV, et al (2009) Origin and evolution of the Notch signalling pathway: an overview from eukaryotic genomes. BMC Evol Biol 9: 249. 19825158 10.1186/1471-2148-9-249 PMC2770060

[2] Richards GS, Degnan BM (2009) The dawn of developmental signaling in the metazoa. Cold Spring Harb Symp Quant Biol 74: 81–90. 19903747 10.1101/sqb.2009.74.028

[3] Andersson ER, Sandberg R, Lendahl U (2011) Notch signaling: simplicity in design, versatility in function. Development 138: 3593–3612. 21828089 10.1242/dev.063610

[4] Mumm JS, Kopan R (2000) Notch signaling: from the outside in. Dev Biol 228: 151–165. 11112321 10.1006/dbio.2000.9960

[5] Ohishi K, Varnum-Finney B, Flowers D, Anasetti C, Myerson D, et al (2000) Monocytes express high amounts of Notch and undergo cytokine specific apoptosis following interaction with the Notch ligand, Delta-1. Blood 95: 2847–2854. 10779430

[6] Struhl G, Greenwald I (1999) Presenilin is required for activity and nuclear access of Notch in Drosophila. Nature 398: 522–525. 10206646 10.1038/19091

[7] Brou C, Logeat F, Gupta N, Bessia C, LeBail O, et al (2000) A novel proteolytic cleavage involved in Notch signaling: the role of the disintegrin-metalloprotease TACE. Mol Cell 5: 207–216. 10882063 10.1016/s1097-2765(00)80417-7

[8] Kramer H (2000) RIPping notch apart: a new role for endocytosis in signal transduction? Sci STKE 2000: pe1. 10.1126/stke.2000.29.pe1 11752592

[9] Kopan R (2002) Notch: a membrane-bound transcription factor. J Cell Sci 115: 1095–1097. 11884509 10.1242/jcs.115.6.1095

[10] Tanigaki K, Nogaki F, Takahashi J, Tashiro K, Kurooka H, et al (2001) Notch1 and Notch3 instructively restrict bFGF-responsive multipotent neural progenitor cells to an astroglial fate. Neuron 29: 45–55. 11182080 10.1016/s0896-6273(01)00179-9

[11] Lutolf S, Radtke F, Aguet M, Suter U, Taylor V (2002) Notch1 is required for neuronal and glial differentiation in the cerebellum. Development 129: 373–385. 11807030 10.1242/dev.129.2.373

[12] Stump G, Durrer A, Klein AL, Lutolf S, Suter U, et al (2002) Notch1 and its ligands Delta-like and Jagged are expressed and active in distinct cell populations in the postnatal mouse brain. Mech Dev 114: 153–159. 12175503 10.1016/s0925-4773(02)00043-6

[13] Zhong W, Jiang MM, Weinmaster G, Jan LY, Jan YN (1997) Differential expression of mammalian Numb, Numblike and Notch1 suggests distinct roles during mouse cortical neurogenesis. Development 124: 1887–1897. 9169836 10.1242/dev.124.10.1887

[14] Cui XY, Hu QD, Tekaya M, Shimoda Y, Ang BT, et al (2004) NB-3/Notch1 pathway via Deltex1 promotes neural progenitor cell differentiation into oligodendrocytes. J Biol Chem 279: 25858–25865. 15082708 10.1074/jbc.M313505200

[15] Zhang Q, Wang C, Liu Z, Liu X, Han C, et al (2012) Notch signal suppresses Toll-like receptor-triggered inflammatory responses in macrophages by inhibiting extracellular signal-regulated kinase 1/2-mediated nuclear factor kappaB activation. J Biol Chem 287: 6208–6217. 22205705 10.1074/jbc.M111.310375 PMC3307302

[16] Wongchana W, Palaga T (2012) Direct regulation of interleukin-6 expression by Notch signaling in macrophages. Cell Mol Immunol 9: 155–162. 21983868 10.1038/cmi.2011.36 PMC4002803

[17] Tsao PN, Wei SC, Huang MT, Lee MC, Chou HC, et al (2011) Lipopolysaccharide-induced Notch signaling activation through JNK-dependent pathway regulates inflammatory response. J Biomed Sci 18: 56. 21843347 10.1186/1423-0127-18-56 PMC3176188

[18] Morga E, Mouad-Amazzal L, Felten P, Heurtaux T, Moro M, et al (2009) Jagged1 regulates the activation of astrocytes via modulation of NFkappaB and JAK/STAT/SOCS pathways. Glia 57: 1741–1753. 19455581 10.1002/glia.20887

[19] Niranjan T, Bielesz B, Gruenwald A, Ponda MP, Kopp JB, et al (2008) The Notch pathway in podocytes plays a role in the development of glomerular disease. Nat Med 14: 290–298. 18311147 10.1038/nm1731

[20] Cao Q, Lu J, Kaur C, Sivakumar V, Li F, et al (2008) Expression of Notch-1 receptor and its ligands Jagged-1 and Delta-1 in amoeboid microglia in postnatal rat brain and murine BV-2 cells. Glia 56: 1224–1237. 18449946 10.1002/glia.20692

[21] Wei Z, Chigurupati S, Arumugam TV, Jo DG, Li H, et al (2011) Notch activation enhances the microglia-mediated inflammatory response associated with focal cerebral ischemia. Stroke 42: 2589–2594. 21737799 10.1161/STROKEAHA.111.614834

[22] Grandbarbe L, Michelucci A, Heurtaux T, Hemmer K, Morga E, et al (2007) Notch signaling modulates the activation of microglial cells. Glia 55: 1519–1530. 17705199 10.1002/glia.20553

[23] Correia SC, Carvalho C, Cardoso S, Santos RX, Placido AI, et al.. (2013) Defective HIF Signaling Pathway And Brain Response To Hypoxia In Neurodegenerative Diseases: Not An “Iffy” Question! Curr Pharm Des. In press. 10.2174/1381612811319380013 23530518

[24] Kolias AG, Guilfoyle MR, Helmy A, Allanson J, Hutchinson PJ (2013) Traumatic brain injury in adults. Pract Neurol 13: 228–235. 23487823 10.1136/practneurol-2012-000268

[25] Basovich SN (2010) The role of hypoxia in mental development and in the treatment of mental disorders: a review. Biosci Trends 4: 288–296. 21248426

[26] Parer JT (1998) Effects of fetal asphyxia on brain cell structure and function: limits of tolerance. Comp Biochem Physiol A Mol Integr Physiol 119: 711–716. 9683410 10.1016/s1095-6433(98)01009-5

[27] Johnston MV (1997) Hypoxic and ischemic disorders of infants and children. Lecture for 38th meeting of Japanese Society of Child Neurology, Tokyo, Japan, July 1996. Brain Dev 19: 235–239. 9187471 10.1016/s0387-7604(96)00561-x

[28] Kaur C, Rathnasamy G, Ling EA (2013) Roles of activated microglia in hypoxia induced neuroinflammation in the developing brain and the retina. J Neuroimmune Pharmacol 8: 66–78. 22367679 10.1007/s11481-012-9347-2

[29] Chen Y, Zheng S, Qi D, Zheng S, Guo J, et al (2012) Inhibition of Notch signaling by a gamma-secretase inhibitor attenuates hepatic fibrosis in rats. PLoS One 7: e46512. 23056328 10.1371/journal.pone.0046512 PMC3463607

[30] Cummins AG, Woenig JA, Donato RP, Proctor SJ, Howarth GS, et al (2013) Notch signaling promotes intestinal crypt fission in the infant rat. Dig Dis Sci 58: 678–685. 23053894 10.1007/s10620-012-2422-y

[31] Saura J, Tusell JM, Serratosa J (2003) High-yield isolation of murine microglia by mild trypsinization. Glia 44: 183–189. 14603460 10.1002/glia.10274

[32] Li P, Lu J, Kaur C, Sivakumar V, Tan KL, et al (2009) Expression of cyclooxygenase-1/-2, microsomal prostaglandin-E synthase-1 and E-prostanoid receptor 2 and regulation of inflammatory mediators by PGE(2) in the amoeboid microglia in hypoxic postnatal rats and murine BV-2 cells. Neuroscience 164: 948–962. 19712723 10.1016/j.neuroscience.2009.08.044

[33] Yao L, Kan EM, Lu J, Hao A, Dheen ST, et al (2013) Toll-like receptor 4 mediates microglial activation and production of inflammatory mediators in neonatal rat brain following hypoxia: role of TLR4 in hypoxic microglia. J Neuroinflammation 10: 23. 23388509 10.1186/1742-2094-10-23 PMC3575244

[34] Cao Q, Li P, Lu J, Dheen ST, Kaur C, et al (2010) Nuclear factor-kappaB/p65 responds to changes in the Notch signaling pathway in murine BV-2 cells and in amoeboid microglia in postnatal rats treated with the gamma-secretase complex blocker DAPT. J Neurosci Res 88: 2701–2714. 20648656 10.1002/jnr.22429

[35] Doyle SL, O'Neill LA (2006) Toll-like receptors: from the discovery of NFkappaB to new insights into transcriptional regulations in innate immunity. Biochem Pharmacol 72: 1102–1113. 16930560 10.1016/j.bcp.2006.07.010

[36] Lehnardt S, Massillon L, Follett P, Jensen FE, Ratan R, et al (2003) Activation of innate immunity in the CNS triggers neurodegeneration through a Toll-like receptor 4-dependent pathway. Proc Natl Acad Sci U S A 100: 8514–8519. 12824464 10.1073/pnas.1432609100 PMC166260

[37] Lehnardt S, Lachance C, Patrizi S, Lefebvre S, Follett PL, et al (2002) The toll-like receptor TLR4 is necessary for lipopolysaccharide-induced oligodendrocyte injury in the CNS. J Neurosci 22: 2478–2486. 11923412 10.1523/JNEUROSCI.22-07-02478.2002 PMC6758325

[38] Kim MY, Park JH, Mo JS, Ann EJ, Han SO, et al (2008) Downregulation by lipopolysaccharide of Notch signaling, via nitric oxide. J Cell Sci 121: 1466–1476. 18411251 10.1242/jcs.019018

[39] Palaga T, Buranaruk C, Rengpipat S, Fauq AH, Golde TE, et al (2008) Notch signaling is activated by TLR stimulation and regulates macrophage functions. Eur J Immunol 38: 174–183. 18085664 10.1002/eji.200636999

[40] Foldi J, Chung AY, Xu H, Zhu J, Outtz HH, et al (2010) Autoamplification of Notch signaling in macrophages by TLR-induced and RBP-J-dependent induction of Jagged1. J Immunol 185: 5023–5031. 20870935 10.4049/jimmunol.1001544 PMC3010732

[41] O'Neill LA, Bowie AG (2007) The family of five: TIR-domain-containing adaptors in Toll-like receptor signalling. Nat Rev Immunol 7: 353–364. 17457343 10.1038/nri2079

[42] Fitzgerald KA, Palsson-McDermott EM, Bowie AG, Jefferies CA, Mansell AS, et al (2001) Mal (MyD88-adapter-like) is required for Toll-like receptor-4 signal transduction. Nature 413: 78–83. 11544529 10.1038/35092578

[43] Wu H, Arron JR (2003) TRAF6, a molecular bridge spanning adaptive immunity, innate immunity and osteoimmunology. Bioessays 25: 1096–1105. 14579250 10.1002/bies.10352

[44] Monsalve E, Perez MA, Rubio A, Ruiz-Hidalgo MJ, Baladron V, et al (2006) Notch-1 up-regulation and signaling following macrophage activation modulates gene expression patterns known to affect antigen-presenting capacity and cytotoxic activity. J Immunol 176: 5362–5373. 16622004 10.4049/jimmunol.176.9.5362

[45] Outtz HH, Tattersall IW, Kofler NM, Steinbach N, Kitajewski J (2011) Notch1 controls macrophage recruitment and Notch signaling is activated at sites of endothelial cell anastomosis during retinal angiogenesis in mice. Blood 118: 3436–3439. 21795743 10.1182/blood-2010-12-327015 PMC3179407

[46] Marignol L, Rivera-Figueroa K, Lynch T, Hollywood D (2013) Hypoxia, notch signalling, and prostate cancer. Nat Rev Urol 10: 405–413. 23712204 10.1038/nrurol.2013.110 PMC5240418

[47] Yonekura S, Itoh M, Okuhashi Y, Takahashi Y, Ono A, et al (2013) Effects of the HIF1 Inhibitor, Echinomycin, on Growth and NOTCH Signalling in Leukaemia Cells. Anticancer Res 33: 3099–3103. 23898065

[48] Lanner F, Lee KL, Ortega GC, Sohl M, Li X, et al (2013) Hypoxia-induced arterial differentiation requires adrenomedullin and notch signaling. Stem Cells Dev 22: 1360–1369. 23379656 10.1089/scd.2012.0259

[49] Zhou ZD, Kumari U, Xiao ZC, Tan EK (2010) Notch as a molecular switch in neural stem cells. IUBMB Life 62: 618–623. 20681026 10.1002/iub.362

[50] Wearing HJ, Owen MR, Sherratt JA (2000) Mathematical modelling of juxtacrine patterning. Bull Math Biol 62: 293–320. 10824431 10.1006/bulm.1999.0152

[51] Lai EC (2004) Notch signaling: control of cell communication and cell fate. Development 131: 965–973. 14973298 10.1242/dev.01074

[52] Wang S, Sdrulla AD, diSibio G, Bush G, Nofziger D, et al (1998) Notch receptor activation inhibits oligodendrocyte differentiation. Neuron 21: 63–75. 9697852 10.1016/s0896-6273(00)80515-2

[53] Valapala M, Hose S, Gongora C, Dong L, Wawrousek EF, et al (2013) Impaired endolysosomal function disrupts Notch signalling in optic nerve astrocytes. Nat Commun 4: 1629. 23535650 10.1038/ncomms2624 PMC3718029

[54] Wilhelmsson U, Faiz M, de Pablo Y, Sjoqvist M, Andersson D, et al (2012) Astrocytes negatively regulate neurogenesis through the Jagged1-mediated Notch pathway. Stem Cells 30: 2320–2329. 22887872 10.1002/stem.1196

[55] Hammerle B, Tejedor FJ (2007) A novel function of DELTA-NOTCH signalling mediates the transition from proliferation to neurogenesis in neural progenitor cells. PLoS One 2: e1169. 18000541 10.1371/journal.pone.0001169 PMC2064965

[56] Heyen JR, Ye S, Finck BN, Johnson RW (2000) Interleukin (IL)-10 inhibits IL-6 production in microglia by preventing activation of NF-kappaB. Brain Res Mol Brain Res 77: 138–147. 10814840 10.1016/s0169-328x(00)00042-5

[57] Koscso B, Csoka B, Selmeczy Z, Himer L, Pacher P, et al (2012) Adenosine augments IL-10 production by microglial cells through an A2B adenosine receptor-mediated process. J Immunol 188: 445–453. 22116830 10.4049/jimmunol.1101224 PMC3384725

[58] Matsuno Y, Coelho AL, Jarai G, Westwick J, Hogaboam CM (2012) Notch signaling mediates TGF-beta1-induced epithelial-mesenchymal transition through the induction of Snai1. Int J Biochem Cell Biol 44: 776–789. 22330899 10.1016/j.biocel.2012.01.021

[59] Oeckinghaus A, Hayden MS, Ghosh S (2011) Crosstalk in NF-kappaB signaling pathways. Nat Immunol 12: 695–708. 21772278 10.1038/ni.2065

[60] Cao Q, Kaur C, Wu CY, Lu J, Ling EA (2011) Nuclear factor-kappa beta regulates Notch signaling in production of proinflammatory cytokines and nitric oxide in murine BV-2 microglial cells. Neuroscience 192: 140–154. 21729740 10.1016/j.neuroscience.2011.06.060

[61] Pistollato F, Rampazzo E, Persano L, Abbadi S, Frasson C, et al (2010) Interaction of hypoxia-inducible factor-1alpha and Notch signaling regulates medulloblastoma precursor proliferation and fate. Stem Cells 28: 1918–1929. 20827750 10.1002/stem.518 PMC3474900

[62] Zheng X, Linke S, Dias JM, Zheng X, Gradin K, et al (2008) Interaction with factor inhibiting HIF-1 defines an additional mode of cross-coupling between the Notch and hypoxia signaling pathways. Proc Natl Acad Sci U S A 105: 3368–3373. 18299578 10.1073/pnas.0711591105 PMC2265116

